# 

**Published:** 1890-02-15

**Authors:** 


SATURDAY, FEBRUARY 15th, 1890.

**Epidemics Past and Present** - - - - - - - 303
**Words of Consolation.**—Man's Wants - - - - - 304
**Village Charities.**—II. Occasional Gifts - - - - - 305
**Lady Dufferin's Fund** - - - - - - - - 306
**A Visit to a Training School for the Deaf.**—From a Travelling Correspondent - - - - - - - 306
**The Doctor's Pulpit.**—Influenza Personally Conducted - 307
**Everybody's Page** - - - - - - - - 309
**Notes and News** - - - - - - - - 310
**Annotations.**—Stitches of Progress—Molokai in Calcutta—A St. Thomas' Dresser - - - - - - - 312
**The Practitioner's Outlook and Retrospect:**
Medicine—Latent Gout—Pernicious Anæmia—Syphillis—Pathology of Chorea—Phthisis - - - - - 313
Surgery—Transplantation of Rabbits' Conjunctiva to the Human Eye—Intestinal Strangulation - - - - 314
Hygiene—Medicine and Socialism - - - - - 315
Therapeutics — Adulteration of Drugs—Treatment of Disease by Electricity - - - - - - - 316
**The Hospital Workshop.**—XXI. The Richmond Union Infirmary—By our Special Commissioner - - - - 316
**Passing Topics.**—"Hugh Woods, M.D.," and the Newspapers 318
**Scraps and Gleanings** - - - - - - - - 318

**EXTRA SUPPLEMENT—THE NURSING MIRROR.**

**En Passant.**—Coventry District Association—Nurses' Co-operation—Thousands of Pounds for the National Pension Fund—Sunderland Nursing Institute—Workhouse Infirmary Association—Kent and Canterbury Institute—Short Items—Registration - - - - - - - lxxxi

**Nursing Lectures.** By Percy Lewis, M.D. II. Chest Diseases - - - - - - - - lxxxii

**Notes from Australia** - - - - - - - lxxxiii

**Everybody's Opinion.**—The Title of Nurse—Nursing in America - - - - - - - - - lxxxiii

**Appointments** - - - - - - - - - lxxxiv

**Notes and Queries** - - - - - - - - lxxxiv

**Notices—Vacancies** - - - - - - - - lxxxiv

**Amusements and Relaxation** - - - - - - lxxxiv

